# Detection of clarithromycin resistance and 23SrRNA point mutations in clinical isolates of *Helicobacter pylori* isolates: Phenotypic and molecular methods

**DOI:** 10.1016/j.sjbs.2021.09.024

**Published:** 2021-09-16

**Authors:** Rawaa A. Hussein, Mushtak T.S. Al-Ouqaili, Yasin H. Majeed

**Affiliations:** a Department of Clinical Laboratory Sciences, College of Pharmacy, University of Anbar, Al-Anbar Governorate, Ramadi, Iraq; b Department of Microbiology, College of Medicine, University of Anbar, Al-Anbar Governorate, Ramadi, Iraq; c Department of Internal Medicine, College of Medicine, University of Anbar, Al-Anbar Governorate, Ramadi, Iraq

**Keywords:** *Helicobacter pylori*, Clarithromycin, PCR-RFLP, Point Mutation

## Abstract

**Background and objectives:**

Peptic ulcer disease, chronic gastritis, and stomach cancer are all caused by *H. pylori*. The most notable drug for the treatment is the antibiotic clarithromycin, which is currently the drug of choice. *H. pylori* clarithromycin resistance has been associated with point mutations in 23srRNA, the most prominent of which are A2143 and A2144G*.* In *H. pylori* bacteria, methylase synthesis, macrolide-inactivating enzyme activity, and active efflux have all been found to be resistance mechanisms. The goal of the study is to determine how resistant *H. pylori* is to clarithromycin and what the minimum inhibitory concentration is for various antimicrobials. Furthermore, gastro-endoscopy will be performed on Iraqi patients to detect the presence of A2143G and A2144G point mutations in *Helicobacter pylori* infections, as diagnosed from the pyloric region and other anatomical regions.

**Methods:**

One hundred fifteen samples were collected from patients strongly suspected of *H. pylori* infection presented for upper gastrointestinal endoscopy at Ramadi Teaching Hospitals and Private Clinics for the period from January 2020 until February 2021. Specimens were cultured on brain heart infusion agar containing various antibiotics and were incubated at 37 °C under microaerophilic conditions. For identification of *H. pylori,* isolates of the biochemical tests and RT-PCR assay were applied. The Epsilometer test was used in the antibiotic susceptibility testing as dependent on the CLSI standard. The Restriction Fragment Length Polymorphism technique was used to determine point mutations.

**Results:**

In total, 55 (47.8%) *Helicobacter pylori* isolates were cultured from the 115 biopsy specimens, among which 16 (29.1%), 38 (69.1%), 20 (36.4%), and 40 (72.7%) revealed some degree of resistance to levofloxacin, clarithromycin, ciprofloxacin, and metronidazole, respectively. The frequency of A2144G and A2143 point mutations were 23 (60.5%) and 19 (50%), respectively.

**Conclusions:**

According to our results*, Helicobacter pylori* showed high resistance to clarithromycin. Our results demonstrate the requirement for antibiotic susceptibility testing and molecular methods in selecting drug regimens.

## 1 Introduction

The bacterium *Helicobacter pylori* (*H. pylori)* is microaerophilic, a curved, Gram-negative bacilli isolated in 1983 from gastric biopsy in patients with gastritis (Charitos et al., 2021). Also, it causes persistent gastric infection by colonization of the human gastric mucosa (Ansari and Yamaoka, 2019). *H. pylori* can cause peptic ulcer disease and chronic gastritis (Vazirzadeh et al., 2020). Furthermore mucosa-associated lymphoid tissue and gastric cancer are associated with this microorganism (Arévalo-Jaimes et al., 2019). For these reasons, the WHO has announced *H. pylori* to be a class I carcinogen. The colonization of *H. pylori* must be removed in patients with peptic ulcers as the prevention of long-term ulcers is a major aim in addition to the acceleration of the healing of such (Reddy and Marsicano, 2018).

The spread of *H. pylori* infection differs across the world depending on geographic area, race, sanitary conditions, and age (Mehata et al., 2021). *H. pylori* infection is mainly acquired in one’s early years. It can persist for the host’s lifetime unless specifically treated (Seia et al., 2012). It is well known that more than half of a percent of the world’s population harbour *H. pylori* in the upper part of their GIT, making it the most highly distributed infection worldwide (Khoder et al., 2019). Antibiotic resistance in *H. pylori* is on the increase, which is concerning because it is one of the leading causes of therapy failure. *H. pylori* resistance is substantially higher in underdeveloped countries than in industrialized countries (Savoldi et al., 2018). Antibiotic resistance in *H. pylori* is usually acquired via chromosomal mutations rather than plasmid acquisition. Other resistance mechanisms have been discovered in bacteria, including methylase production, macrolide-inactivating enzyme activities, and active efflux (Agudo et al., 2010). Vertically transmitted point mutations in the DNA are the main cause of *H. pylori* resistance (Zhang et al., 2020).

A point mutation is a form of genetic mutation in which one of the DNA sequence’s base pairs is changed, either by insertion or deletion. A point mutation that occurs as a result of mutagens can be physical, such as X-rays and UV radiation, or due to chemical species, which can alter base pairs and DNA structure (Mobley, et al., 2001).

A2144G, A2143G, and A2142C are the most prevalent mutations. A2115G, G2141A, C2147G, T2190C, C2195T, A2223G, and C2694A mutations have also been discovered, but their significance in clarithromycin resistance is unclear (Zhang et al., 2020). The most common mutations in 23SrRNA gene are A2143G or A2144G in resistant *H. pylori* strains (Matta et al., 2018).

In spite of the sensitivity of *H. pylori* to various drugs *in vitro*, its elimination is difficult *in vivo*. This is believed to be due to the deactivation of antibiotics in the acidic pH of the stomach (Vala et al., 2016). Generally, triple therapy consists of a proton pump inhibitor together with double antibiotics such metronidazole and amoxicillin or clarithromycin, and is used for treatment of *H. pylori* infection (Ong et al., 2019). Due to an increase in resistance of *H. pylori* to clarithromycin, the efficacy of this regimen has dramatically decreased (Huang et al., 2017).

Clarithromycin interacts with the peptidyl transferase region of domain V of the 23S rRNA subunit, decreasing bacterial protein synthesis and suppressing bacterial ribosome activity, hence 23S rRNA was chosen for amplification and point mutation detection (Marques et al., 2020). Point mutations in the 23S rRNA gene and post-transcriptional methylation of the 23S rRNA region have been found to cause a change in ribosome structure, decreasing clarithromycin affinity and resulting in bacterial resistance to the antibiotic (Zhang et al., 2020). This antibiotic has an option for elimination of *H. pylori* as compared to other macrolides due to its stability in acid pH and its good absorption in the stomach (Marques et al., 2020). Clarithromycin resistance is responsible for clarithromycin treatment failure in over 50% of such cases (Yoon et al., 2014a, Yoon et al., 2014b). High outpatient consumption of clarithromycin, particularly in the treatment of respiratory tract disorders, is one of the key contributors to *H. pylori* strains’ clarithromycin resistance (Klesiewicz et al., 2014).

As a result, in any region, determining antibiotic sensitivity before using clarithromycin to treat *H. pylori* infection is critical (Vala et al., 2016). Approved antibiotic susceptibility testing (AST) methods against bacteria, such as agar dilution, E-test, and disk diffusion, make this possible. The gold standard approach is agar dilution, however it is time consuming and costly. As a result, E-test or disk diffusion procedures could be used in place of agar dilution The E-test is a simple and straightforward method for measuring the minimal inhibitory concentration (MIC) of a range of antibiotics, however it is more costly (Vala et al., 2016). The underlying aim of the study is to detect the resistance of *H. pylori* to clarithromycin and the minimal inhibitory concentration for selected antimicrobials and, in addition, to detect the occurrence of A2143G and A2144G point mutations in 23SrRNA of clarithromycin-resistant *H. pylori* isolates from a sample in Anbar, Iraq.

## 2 Patients study and techniques

### 2.1 Specimen collection

In total, 115 patients who submitted for routine upper gastrointestinal endoscopy at Ramadi Teaching Hospitals during the period from January 2020 until February 2021 constituted the sample. The patients included 80 (69.6%) males and 35 (30.4%) females with ages ranging from 17 to 69 years. The clinical diagnoses based on endoscopy included antral gastritis (n = 53), combined gastritis and duodenitis (n = 2), duodenitis (n = 7), gastric tumour, and adenocarcinoma (n = 1), hiatus hernia (n = 4), combined gastric and duodenal ulcers (n = 19), esophagitis (n = 1), and patients with dyspepsia (n = 28) (Table 1). The exclusion criteria were applied to patients who had received H2 receptor blockers, antimicrobial therapy, PPI, and/or non-steroid anti-inflammatory drugs one month pre-endoscopy. Subjects with the following clinical conditions were also excluded from the study: cirrhosis, nephropathy in critical stages, and pregnancy. Information about demographic and socioeconomic factors, and the personal treatment histories of the included patients was already reported in a questionnaire. The gastric biopsy samples obtained from the antrum and corpus of the stomach during routine endoscopy by an expert clinician (gastroenterologist) were placed in sterile tubes containing brain heart infusion broth medium and 5% of foetal bovine serum for transportation. Further, the results of an invasive rapid urease test (RUT) were found using a urea agar slant tube, RT-PCR amplification of 16srRNA using the thermal cycler (Sacace - Italy) with a RT-PCR kit for qualitative detection of *H. pylori* ((Sacace- Italy), UBT using a HUBT-20p *H. pylori* detector (HEADWAY, China) using^14^ a C-urea, 99-atom%^14^ C-labelled urea capsule. SAT was performed using the *H. pylori* Ag Rapid Test CE (CTK - Biotech, USA), whilst cagA-IgG was performed using a commercial Human *H. Pylori* Cytotoxin-Associated Gene A Protein IgG (HP-CagA-IgG) ELISA Kit (CUSABIO, USA) by ELISA system (Human, Germany) (Hussein et al., 2021).

**Table 1:** Guideline characters for the main topics regarding the study patients and their diseases.

**Items**	**Findings**
Number of patients (study isolates)	115 (55)
Age	17–69 years
Men: women (men: women %)	80: 35 (69.6%: 30.4%)
**Disease**	
Antral gastritis: Duodenitis: Esophagitis: Hiatus-Hernia	53:7:1:4
Combined gastritis/Duodenitis: GDU	2:19
Gastric tumour	1
Patients with dyspepsia	28

*GDU, combined gastric and duodenal ulcer.

### 2.2 Ethic’s committee

All study techniques that involved patients were approved by the Ethical Approval Committee, University of Anbar, Ramadi, Iraq (approval number 122, November 20, 2019) in accordance with the Declaration of Helsinki. Informed written consent was provided by all patients (or their parents) participating in the study.

### 2.3 Strains Isolation, identification and storage

Samples were cultivated on brain heart infusion agar (Oxoid, UK) consisting of 5% foetal bovine serum (Capricorn, South America), 7% horse blood, and antibiotics including nystatin, nalidixic acid, and vancomycin. The cultures were then incubated overnight (with specific conditions regarding microaerophilic conditions and saturated humidity: < 0.1 oxygen within 2.5 h and 7–15% CO_2_ within 24 hr. for 5–7 days (Anaerocult A; Darmstadt, Germany)) (Amin et al., 2019). Colonies of *H. pylori* was diagnosed and well identified bacteriologically. In addition, *H. pylori* isolates were confirmed by reverse transcription polymerase chain reaction amplification. *H. pylori* preservation were stored in brain heart infusion broth containing 10% foetal bovine serum and 15% glycerol at −20 °C. These stock solutions were thawed and sub-cultured for the study experiments (Amin et al., 2019).

### 2.4 Determination of antibiotic MICs using the E-Test

The MIC values of clarithromycin, levofloxacin, ciprofloxacin, and metronidazole against *H. pylori* isolates were confirmed with epsilometer strips (E-test, Ezy MIC™ Strips, HiMedia Laboratories, India). This test is performed and standardized based on criteria laid down by the appropriate Clinical Laboratory Standard Institute protocol. *H. pylori* isolates were sub-cultured on brain heart infusion agar (Oxoid, England) supplemented with 7% of horse blood, 5% foetal bovine serum, and dent supplement (vancomycin, nystatin, and nalidixic acid) for 72 h. A suspension of the bacteria was prepared equivalent to the McFarland turbidity standard (9x10^8^ CFU/mL; turbidity, 3 McFarland) and inoculated to Mueller-Hinton agar (Oxoid, England) containing 7% horse blood. The E test strips were placed on the Mueller-Hinton agar after drying the surface of the medium. They were incubated under microaerophilic conditions at 37 °C for 72 h. Resistant breakpoints of MICs for clarithromycin, levofloxacin, ciprofloxacin, and metronidazole, were defined as ≥ 8, ≥ 8, ≥ 4, and ≥ 32 μg/mL, respectively, whereas the susceptible strains had MICs for clarithromycin, levofloxacin, ciprofloxacin, and metronidazole, ≤ 2, ≤ 2, ≤ 1, and **≤** 8 μg/mL, respectively (Amin et al., 2019, Al-Qaysi et al., 2020).

### 2.5 **Molecular technique:-** (Sambrook et al., 1989)

a) Extraction of DNA:

DNA was extracted using SaMag Tissue DNA extraction kits (Sacace, Italy) using the SaMag-12 automatic nucleic acid extraction system for the extraction of genomic DNA (Samaga, Cepheid, Italy). For the RFLP technique, the SaMag Bacterial DNA extraction kit (Sacace, Italy) was used to extract the nucleic acid from 38 (69.1%) *H. pylori* isolates which were resistant to clarithromycin. The extracted DNA was stored at a temperature of −20 °C (Vaziri et al., 2013). A QuantusTM Fluorometer (Promega, USA) was used to determine the concentration of extracted DNA to detect the goodness of the sample for further applications (Khalaf and Al-Ouqaili, 2018, Al-Ouqaili et al., 2020).

b) Quantitative real-time PCR (qRT-PCR):

This was achieved using a thermal cycler (Sacace - Italy) with a Real-Time PCR kit for the qualitative detection of *H. pylori* ((Sacace, Italy). PCR conditions consisted of 1 cycle of 15 min at 95 °C. The cycling programme consists of 45 denaturation cycles for 10 s at 95 °C followed by annealing for 30 s at 60 °C, and an extension of 10 s at 72 °C.

c) 23SrRNA amplification and PCR-RFLP

DNA was extracted from *H. pylori* isolates using SaMag Bacterial DNA extraction kits (Sacace, Italy) which act as a template for amplifying a 425 bp fragment of 23SrRNA gene peptidyl transferase. Oligonucleotide primers (sense, 5́-CCACAGCGATGTGGTCTCAG-3́; antisense, 5́-CTCCATAA- GAGCCAAAGCCC-3́) were used to investigate the mutations in the 23S rRNA gene that emerged in clarithromycin resistance. The polymerase chain reaction followed a program of initial denaturation at 94 °C for 3 min, followed by 35 cycles of denaturation at 94 °C for 1 min, annealing for 1 min at 55 °C, an extension of 1 min at 72 °C and a final extension for 7 min at 72 °C to amplify the 23srRNA. INtRON is the Maxime PCR PreMix Kit (Intron, Korea), which includes 5 U/µl i-Taq DNA polymerase, 2.5 mM DNTPs, 1X reaction buffer (10X), and 1X gel loading buffer. The PCR reaction was achieved using a final volume of 25 µl containing 5 µl Taq PCR PreMix, 10 picomols/µl (1 µl) forward and reverse primer, 1.5 μl genomic DNA as a template, and 16.5 µl distilled water. Red Safe Nucleic Acid Staining (Intron, Korea) was selected to stain the PCR product (425 bp) through electrophoresis using 1.5% agarose (Amin et al., 2019).

d) PCR-RFLP and detection of point mutations

The PCR-RFLP assay was performed to identify point mutations in clarithromycin-resistant strains of *H pylori*. Amplicons (425 bp each) of the 23S rRNA gene were digested with either *BsaI* enzymes (BioLabs, USA) for 30 min at 37 °C or *BbsI* (BioLabs, USA) for 30 min at 37 °C to detect the A2143G and A2144G mutations, respectively, as shown in Table 2. Digested fragments were separated on a 2.5% agarose gel and viewed under UV light (Vilber Lourmat, France). A2144G and A2143G mutations in clarithromycin resistance strains were identified according to fragment numbers and size (Yoon et al., 2014a, Yoon et al., 2014b).

**Table 2:** Laboratory parameters for restriction enzyme digestion including products’ size and thermal profiles according to the study point mutations.

**Type of Mutations**	**Study Enzyme**	**Thermal profile**	**Size of products**	**Reference**
**A2144G**	BsaI	37 °C (30 min)	304 bp and 101 bp	(Klesiewicz et al., 2014)
**A2143G**	BbsI	37 °C (30 min)	332 bp and 93 bp	

## 3 Results

**Fig. 1:**
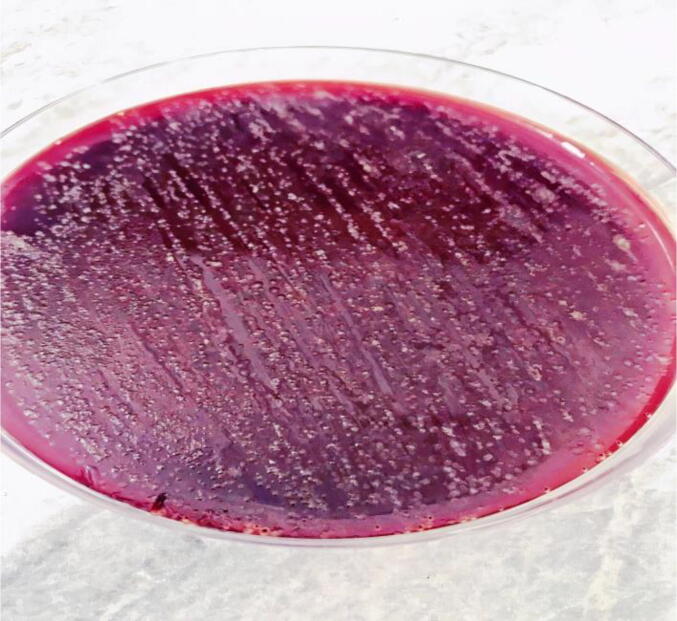
Culture of *H. Pylori-*positive isolates.

**Fig. 2:**
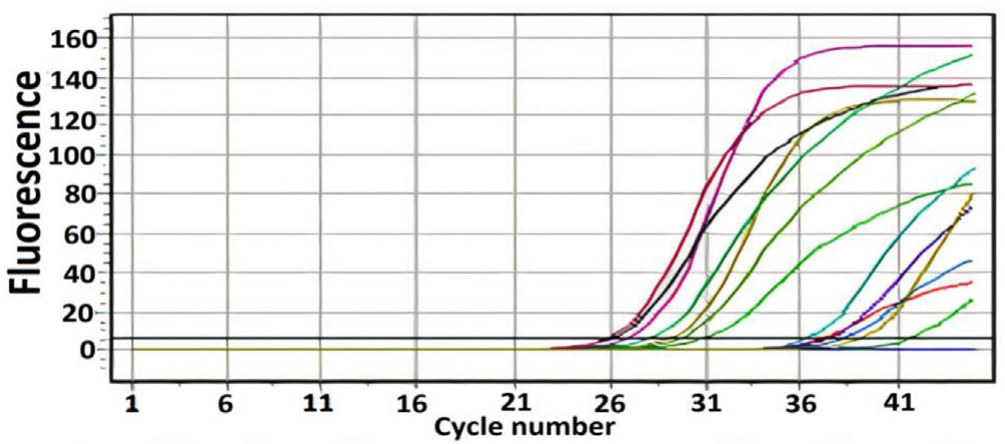
RT-PCR positive result for *H. Pylori.*

In total, 55 (47.8%) *H. pylori* cultures were obtained from 115 biopsies. A total of 41 (51.3%) and 14 (40%) of the isolates were collected and diagnosed from both males and females. The males and females were within the age ranges of 18–69 and 17–66, respectively. The patients had a mean age of 36.53 ± 14.544. The macroscopic cultural characteristics of *H. pylori* on the culture plate were confirmed by using all requested biochemical and molecular diagnostic tools, which include urease, oxidase, catalase, and the reverse transcription polymerase chain reaction, where the latter was performed to amplify and detect 16SrRNA (Fig. 1, Fig. 2). The endoscopy-based clinical diagnoses included antral gastritis, combined gastritis and duodenitis, duodenitis, gastric tumour or adenocarcinoma, hiatus hernia, combined gastric and duodenal ulcers, esophagitis, and patients with dyspepsia.

### 3.1 Detection of antibiotics’ minimal inhibitory concentrations (MIC)

**Fig. 3:**
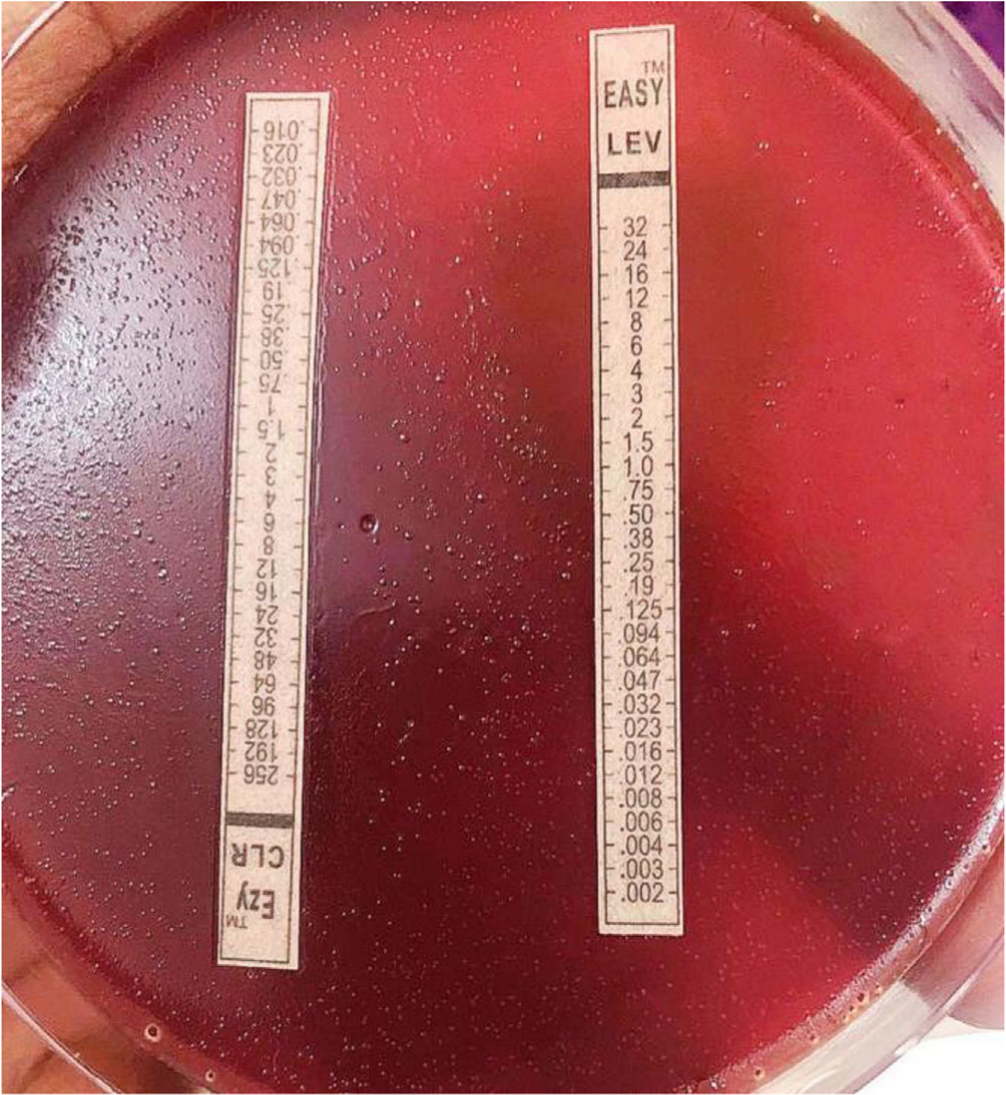
*H. pylori* strain sensitive to levofloxacin and resistant to clarithromycin.

Clarithromycin, levofloxacin, ciprofloxacin, and metronidazole resistance was found in 38 (69.1%), 16 (29.1%), 20 (36.4%), and 40 (72.7%) of the 55*H. pylori* strains, respectively. (Table 3). Additionally, the antibiotic MICs based on the E-test are represented in Fig. 3. The clarithromycin, levofloxacin, ciprofloxacin, and metronidazole MIC values ranged from 0.25 to 128 μg/mL, 0.064 to 32 μg/mL, 0.094 to 64 μg/mL, and 0.19 to 256 μg/mL, respectively.

**Table 3:** The prevalence of resistance to antimicrobial agents against Iraqi *H. pylori* isolates.

**Antibiotics**	**% resistance (Number of the resistant isolates per total isolates)**
**clarithromycin**	69.1 (38/55)
**levofloxacin**	29.1 (16/55)
**ciprofloxacin**	36.4 (20/55)
**metronidazole**	72.7 (40/55)

### 3.2 The multidrug resistance study isolates

44 of 55 strains (80%) were resistant to two antimicrobial agents (revealing MDR) (Table 4). Clarithromycin and metronidazole resistance were the most prevalent multidrug-resistant isolates (54.5%). Resistance to more than two drugs were observed in nine (16.4%) and four (7.3%) strains, respectively.

**Table 4:** Prevalence of multi-drug resistant *H. pylori.*

**Number of resistant antibiotics**	**multidrug resistance type**	**No. of resistant isolates**
2	CLA + CIP ClA + MET CLA + LEV LEV + CIP CIP + MET	3 24 1 2 1
3	CLA + LEV + MET CLA + CIP + MTE LEV + CIP + MET	1 2 6
4	CLA + LEV + CIP + MET	4
**Total**		44 (80%)

### 3.3 Amplification of the 23S rRNA in clarithromycin-resistant strains

**Fig. 4:**
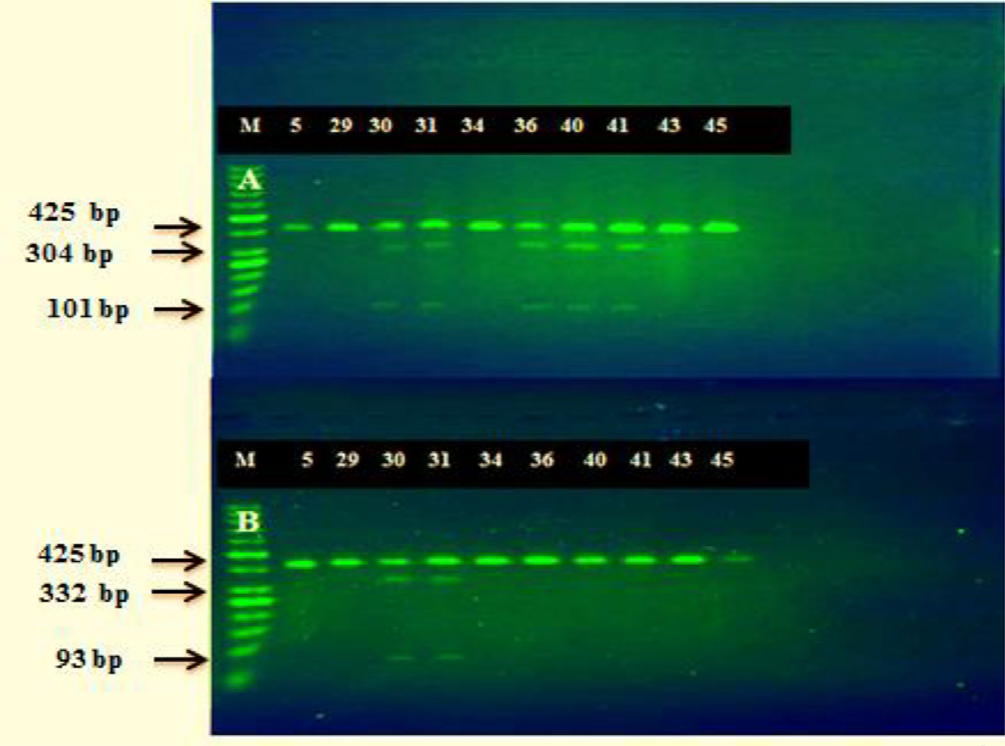
RFLP analysis of 23S rRNA amplicons (425 bp) digested with: (Part A) *BsaI* enzyme (digestion products of 304 and 101 bp for A2144G mutants); (Part B) *BbsI* enzyme (digestion products of 332 and 93 bp for A2143G mutants); lines 5–45: clinical *H. pylori* isolates; lines 30, 31, 36, 40, 41: A2144G *H. pylori* mutants; Lines 30, 31 (double mutation) A2143G *H. pylori* mutants; lines (5, 29, 34, 43, 45) clarithromycin-resistant isolate with negative results for both assayed mutations; M = Molecular Weight Marker (50 bp Intron, Korea).

The PCR technique was performed to amplify a fragment (425 bp) from the PT variable domain of 23srRNA to detect point mutations in clarithromycin-resistant isolates. The *BsaI* and *BbsI* endonuclease enzymes influence the PCR product. The PCR product of the strains containing the A2144G mutation produced 304-bp and 101-bp fragments if digested with *BsaI*. The *H. pylori* strains containing the A2143G mutation produced 93-bp and 332-bp fragments if digested with *BbsI*, as per Fig. 4. The A2144G mutation was found in 23 (60.5%) of the 38 clarithromycin-resistant *H. pylori* strains studied, whereas the A2143G mutation was found in 19 (50%) and the A2144G and A2143G double mutation was found in 9 (23.7%).The amplified products were not digested by *BsaI* and *BbsI* (15; 39.5%) (19; 50%), respectively (Table 6), which did not contain any of the above mentioned mutations. (The MICs for the A2144G mutant strains ranged from 8 to 32 μg/mL. The MICs for the A2143G mutant strains ranged from 32 to 128. The MICs for the double mutant strain were 16 μg/mL. The MICs for non-restricted strains were relatively low (MICs of 8 μg/mL) (Table 6).

### 3.4 Relationship between the type of mutation and the level of clarithromycin resistance

In total, of the 38*H. pylori*-resistant strains, 14 (41.2%) A2144G *H. pylori* mutants had a MIC ≤ 32 μg/mL, six (17.6%) A2143G *H. pylori* mutants had a MIC ≤ 32 μg/mL , nine (26.5%) double mutant *H. pylori* had a MIC ≤ 32 μg/mL and 5 (14.7%) isolates that lacked either mutation and whose basis for clarithromycin resistance remains undetermined, had a MIC ≤ 32 μg/mL, while only 4 (100%) A2143G *H. pylori* mutants had a MIC > 32 μg/mL (Table 5).

**Table 5:** Association between MICs of clarithromycin (mg/L) and type of mutations in the 23S rRNA of clarithromycin-resistant *H. pylori* strains.

**Mutation**	**Number (%) of strains**		
	**MIC ≤ 32 mg/L** **n = 34**	**MIC > 32 mg/L** **n = 4**	**Total** **n = 38**
A2143G	6 (17.6%)	4	10 (26.3%)
A2144G	14 (41.2%)	0	14 (36.8%)
Double mutation (A2144G, A2143G)	9 (26.5%)	0	9 (23.7%)
Undetermined (NR)	5 (14.7%)	0	5 (13.2%)

**Table 6:** PCR-RFLP method and mutations in 38 clarithromycin-resistant strains.

**Isolates**	**Diagnosis**	**Age/sex**	**RFLP**	**MIC (μg/mL)**	**Mutation**
4	Antral gastritis	31/M	*BsaI, BbsI*	8	NR, NR
5	GDU	20/F	*BsaI, BbsI*	8	NR, NR
6	Antral gastritis	34/M	*BsaI, BbsI*	16	A2144G, A2143G
7	GDU	45/M	*BsaI, BbsI*	16	A2144G, A2143G
10	Antral gastritis	20/M	*BsaI, BbsI*	16	A2144G, A2143G
11	GDU	56/M	*BsaI, BbsI*	16	A2144G, A2143G
22	Antral gastritis	30/M	*BsaI, BbsI*	32	NR, A2143G
24	Antral gastritis	24/M	*BsaI, BbsI*	8	A2144G, NR
25	GDU	25/M	*BsaI, BbsI*	32	NR, A2143G
26	Duodenitis	18/M	*BsaI, BbsI*	8	NR, NR
28	Antral gastritis	35/M	*BsaI, BbsI*	16	A2144G, NR
29	GDU	69/M	*BsaI, BbsI*	128	NR, A2143G
30	Duodenitis	31/M	*BsaI, BbsI*	16	A2144G, A2143G
31	GDU	32/F	*BsaI, BbsI*	16	A2144G, A2143G
34	Antral gastritis	38/F	*BsaI, BbsI*	8	NR, NR
36	Duodenitis	30/M	*BsaI, BbsI*	16	A2144G, NR
40	Antral gastritis	20/M	*BsaI, BbsI*	16	A2144G, NR
41	Antral gastritis	52/M	*BsaI, BbsI*	16	A2144G, NR
43	Antral gastritis	25/F	*BsaI, BbsI*	64	NR, A2143G
45	Dyspepsia	23/F	*BsaI, BbsI*	8	NR, NR
51	Antral gastritis	25/M	*BsaI, BbsI*	32	NR, A2143G
59	GDU	18/M	*BsaI, BbsI*	64	NR, A2143G
60	GDU	26/M	*BsaI, BbsI*	32	NR, A2143G
61	Antral gastritis	31/F	*BsaI, BbsI*	8	A2144G, NR
62	Antral gastritis	36/M	*BsaI, BbsI*	16	A2144G, NR
63	Antral gastritis	26/M	*BsaI, BbsI*	32	A2144G, NR
64	GDU	32/M	*BsaI, BbsI*	16	A2144G, NR
65	GDU	69/M	*BsaI, BbsI*	16	A2144G, A2143G
66	Antral gastritis	45/F	*BsaI, BbsI*	16	A2144G, A2143G
67	Antral gastritis	32/M	*BsaI, BbsI*	16	A2144G, NR
68	Antral gastritis	50/M	*BsaI, BbsI*	16	A2144G, NR
69	Deuodenitis	20/M	*BsaI, BbsI*	16	A2144G, NR
70	Antral gastritis	31/M	*BsaI, BbsI*	16	A2144G, NR
71	GDU	19/F	*BsaI, BbsI*	16	A2144G, NR
72	GDU	30/M	*BsaI, BbsI*	32	NR, A2143G
73	Antral gastritis	18/M	*BsaI, BbsI*	64	NR, A2143G
74	GDU	20/F	*BsaI, BbsI*	32	NR, A2143G
83	GDU	36/M	*BsaI, BbsI*	16	A2144G, A2143G

* GDU, combined gastric and duodenal ulcer; MIC, minimal inhibitory concentration; NR, non-restriction; RFLP, restriction fragment length polymorphism.

## 4 Discussion

The resistance of *H. pylori* to antibiotics has been observed worldwide. Iraq is among those countries in which resistance to the drug of choice is spreading. Clarithromycin is used largely in *H. pylori* treatment. Therefore, the increased resistance has become a major issue in the eradication of study bacterium (Goderska, 2018) . *H. pylori* resistance to clarithromycin is predominantly related to the point mutations in the peptidyl transferase-encoding region of the V domain of the 23S rRNA gene. The most prevalent point mutations responsible for this process are A2143G, A2142G, and A2142C (Klesiewicz et al., 2014). Norway (5.9%) has the lowest levels of clarithromycin resistance in Europe, whereas Spain (32.01%) and Portugal have the highest (42.35%). *H. pylori* resistance decreased from 36.65% in 2009 to 24.38% in 2014 according to European research conducted at six-year intervals, whilst India (58.8%) and China (58.8%) both saw high levels of clarithromycin resistance in the Asian regions (Ghotaslou, 2015). In Pakistan and Iran, resistance has been estimated to be 47.8% and 31.7%, respectively (Rasheed et al., 2014). Clarithromycin resistance has increased in several countries in recent years as a result of the extensive use of clarithromycin for respiratory infections within the general public, particularly in children, and there is a link between outpatient use of long-acting macrolides and clarithromycin resistance (Ghotaslou, 2015). Epidemiological factors and inadequate therapy leading to misuse of antibiotics may play a vital role in the variation of resistance to clarithromycin across the world (Aslam et al., 2018). Depending on the E-test method used, the isolated *H. pylori* strains showed resistance to clarithromycin in 38 (69.1%) cases in this study, which is close to the values observed by Abdollahi et al. (2011) in Iran (69.3%). Our results were higher than those of de Francesco *et al*. (2010) from Italy (51.2%), Amin et al. (2019) from Iran (53.4%), and Arenas et al. (2019) from Chile (26%). However, the results are largely inconsistent with the studies by Lottspeich et al., (2007) from Germany (13.3%), Tani and Ndip (2013) from South Africa (15.4%), Jaka et al., (2018) from Africa (29.2%) and Wang et al., (2019) from China (31.0%).

Klesiewicz et al. (2014) reported that different types of mutations are associated with different MIC values. The relationship between the MICs and the type of mutation for the 38 analysed *H. pylori* isolates is shown in Table 5. Versalovic and associates' (1997) observations are inconsistent with those from our study, in which MIC values exceeding 32 mg/L were defined as having high-level resistance to clarithromycin. Our results showed that all of the A2144G *H. pylori* mutants demonstrated low MICs to clarithromycin (MICs ≤ 32 mg/L), while in the A2143G mutants we observed strains of both phenotypes, that is, with high- or low-level resistance. However, the results of our study demonstrated that the strains with the A2144G mutation had lower average MICs than strains with the A2143G mutation (8 mg/L and 32 mg/L, respectively). These results are in line with those found by other researchers who concluded that the A2144G point mutation was correlated with lower clarithromycin MICs than A2143G (Klesiewicz et al., 2014).

For the first time, Versalovich and associates stated that point mutations in the 23srRNA variable region are related to *H. pylori* resistance to clarithromycin (Abdollahi et al., 2011). Point mutations, which result in A to G transitions in the 23 s rRNA sequence, can be found at positions 2143 and 2144 and which have been subsequently confirmed by other investigators (Yoon et al., 2014a, Yoon et al., 2014b, Amin et al., 2019). In this study, A2144G and A2143G mutations were found in *H. pylori* clarithromycin-resistant strains, based on the PCR-RFLP assay; among the 38 (69.1%) clarithromycin-resistant *H. pylori* strains included in our study, 23 (60.5%) of the isolates carried the A2144G mutation, 19 (50%) carried the A2143G mutation, and 9 (23.7%) carried the double mutation (A2144G and A2143G). PCR products were not digested by *BsaI* and *BbsI* were (15; 39.5%) (19; 50%), respectively, which did not contain any of the above mentioned mutations. Therefore, our research confirms the results reported by several other authors that the predominant mutations responsible for clarithromycin resistance in *H. pylori* are A2144G and A2143G (Yoon et al., 2014a, Yoon et al., 2014b, Vazirzadeh et al., 2020). The majority of isolates (60.5%) contained the A2144G mutation, whereas the A2143G mutation was found in half (50%), where our results were consistent with Álvarez et al. (2009) from Colombia, Yoon et al., 2014a, Yoon et al., 2014b from Korea, Klesiewicz et al. (2014) from Poland, and Vazirzadeh et al. (2020) from Iran. Therefore, there are geographical variations in clarithromycin resistance, which highlights the significance of identifying regional patterns of resistance in *H. pylori* for the selection of proper treatment. Furthermore, the clarithromycin resistance is attributed to the efflux system in *H. pylori*, which forces the bacteria to extrude macrolides. Our PCR-RFLP analysis also showed the occurrence of five (14.7%) isolates without any digestion with *BsaI* and *BbsI*. Therefore, resistance of these isolates to clarithromycin might be associated with other less common mutations or with the efflux mechanism (Klesiewicz et al., 2014).

In our study, 55*H. pylori* strains were tested; 3.7% were susceptible to all, and 96.3% were resistant to at least one of the tested antibiotics. Multi-resistant strains explained as many as 80% of cases and included those resistant to two (metronidazole and clarithromycin, or clarithromycin and ciprofloxacin, or clarithromycin and levofloxacin, or levofloxacin and ciprofloxacin, or ciprofloxacin and metronidazole, or three (clarithromycin, levofloxacin, and metronidazole, or clarithromycin, ciprofloxacin and metronidazole, or levofloxacin, ciprofloxacin and metronidazole) or four (clarithromycin, levofloxacin, ciprofloxacin and metronidazole). This rate was higher than that (16.5%) for *H. pylori* isolated from Korean patients (Yoon et al., 2014a, Yoon et al., 2014b) and from Polish patients (29%) (Bińkowska et al., 2018). Our study showed a high resistance to metronidazole that was in line with a study in Eastern and Central European countries, reaching almost 50%, whilst resistance to clarithromycin is as high as 30% and still increasing, contributing to the failure of first-line therapy in approximately 70% of patients (Mégraud, 2017). Such a high resistance to clarithromycin as that observed in Poland results mainly from excessive consumption of antibiotics, especially from the group of macrolides, and their widespread use to treat respiratory tract infections. With regard to levofloxacin, our results showed resistance to such was very low (29.1%) which is very close the rates observed by Karczewska et al., 2014, Korona-Glowniak, 2019.

## 5 Conclusions

According to our results, *H. pylori* showed high resistance to clarithromycin. Our results demonstrated the necessity for antibiotic susceptibility testing and molecular methods when selecting drug regimens. The correlation between the resistance of *H. pylori* to clarithromycin and 23srRNA point mutations is clearly high. In addition, *H. pylori* resistance to clarithromycin is both widespread and considerable, resulting in the reduced potencies seen in studies of antimicrobial agents. However, new antimicrobial resistance studies should be handled periodically and regionally in Iraq to provide information that may help to monitor effective eradication programmes. The PCR-RFLP method reduces the time required for the determination of clarithromycin resistance by about four days compared to phenotypic methods of susceptibility testing. Non-restricted isolates were also discovered during our PCR-RFLP study. Resistance to clarithromycin in these isolates could be associated with other, less prevalent mutations or the efflux mechanism. As a result, more research in this area is clearly required. These mechanisms could be determined at some future point via PCR-RFLP, RT-PCR, or sequencing. Our observations revealed that patients showed considerable clarithromycin resistance and that *H. pylori* can have dual resistance. This shows that existing *H. pylori* infection management in this part of Iraq should be adjusted to account for the high prevalence of *H. pylori* resistance to clarithromycin. Clarithromycin-based triple therapy should not be utilized in the attempt to eradicate *H. pylori*.

## Declaration of Competing Interest

The authors declare that they have no known competing financial interests or personal relationships that could have appeared to influence the work reported in this paper.
